# CAT‐BLAST: Engineered bacteria for robust targeting and elimination of cancer‐associated fibroblasts

**DOI:** 10.1002/imt2.70102

**Published:** 2026-01-05

**Authors:** Mengdi Xu, Ehsan Hashemi, Hui Gao, Qumar Zaman, Yi Ma, Jufang Wang, Wenjun Mao, Zhuobin Liang

**Affiliations:** ^1^ School of Biology and Biological Engineering South China University of Technology Guangzhou China; ^2^ Institute of Molecular Physiology Shenzhen Bay Laboratory Shenzhen China; ^3^ Department of Cardiothoracic Surgery The Affiliated Wuxi People's Hospital of Nanjing Medical University Wuxi Jiangsu China

## Abstract

Cancer‐associated fibroblasts (CAFs) construct a protective stromal barrier that promotes tumor growth and resistance to therapy. To dismantle this, we developed CAT‐BLAST, an engineered bacterial platform designed to potently eliminate these cells. We engineered a safe, high‐expression *E. coli* chassis, arming it with a FAP‐specific synthetic adhesin for precise CAF targeting and the ability to secrete the ClyA cytotoxin to induce apoptosis in both CAFs and adjacent tumor cells. This platform demonstrated robust, FAP‐specific targeting across diverse human tumor xenograft models and achieved significant tumor suppression in both murine colorectal cancer and melanoma.

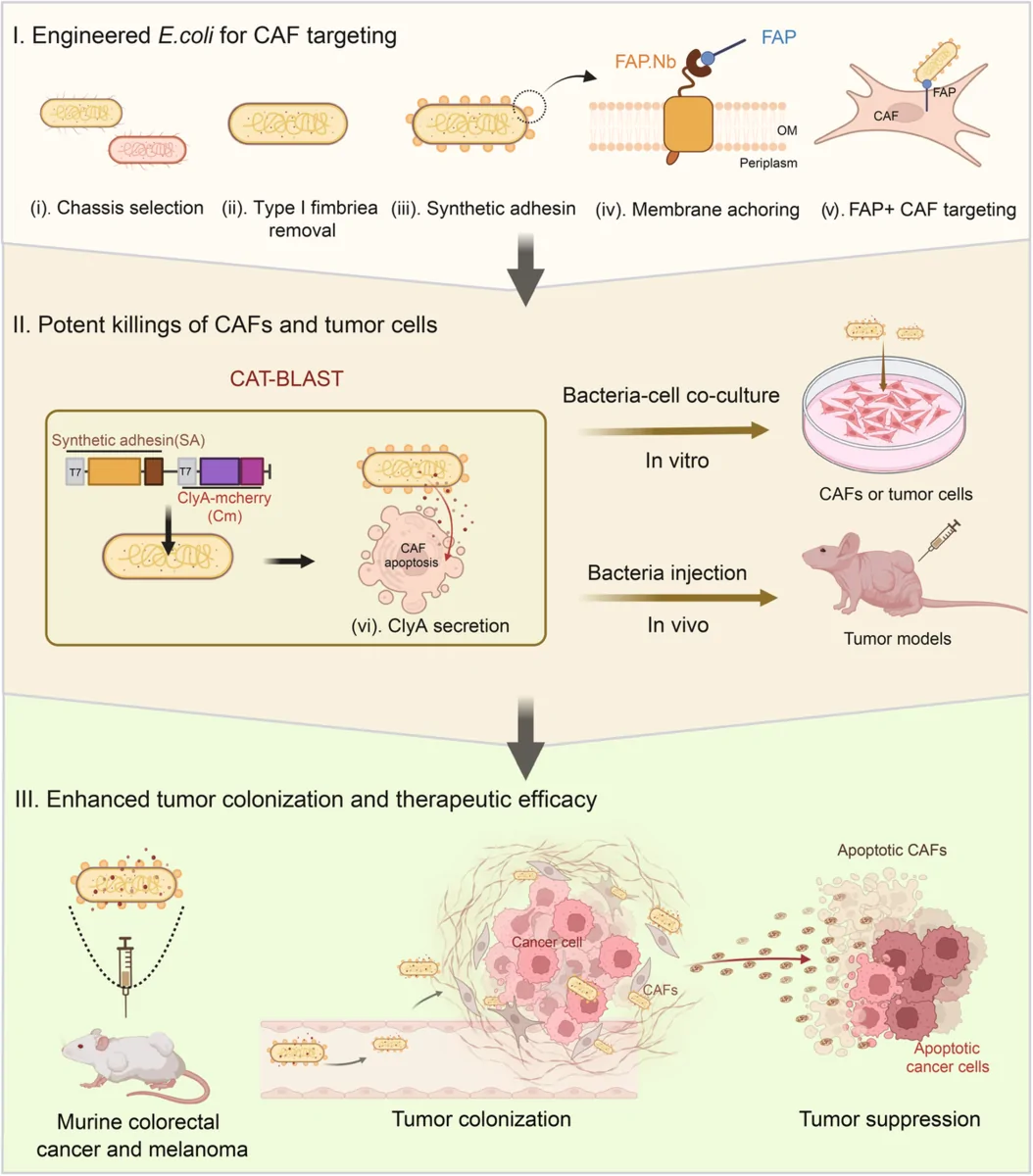

To the Editor,

The tumor microenvironment (TME) is a major driver of cancer progression and therapy resistance. Within the TME, cancer‐associated fibroblasts (CAFs) are a primary component of the tumor stroma, where they form a dense, protective barrier. This barrier actively promotes tumor growth, facilitates metastasis, and limits the efficacy of many cancer therapies, including immunotherapy and chemotherapy [1, 2, 3].

Targeting CAFs for depletion has therefore emerged as a promising therapeutic strategy [4, 5]. Fibroblast activation protein (FAP), a peptidase highly expressed on CAFs across numerous cancers but largely absent in healthy tissues, represents an ideal target for this approach [6]. However, existing FAP‐targeted modalities, such as CAR‐T cells and antibody‐based drugs, have been hampered by significant challenges, including insufficient tumor penetration, limited persistence, and potential off‐tumor toxicity [7, 8, 9, 10, 11]. This highlights an urgent need for novel strategies that can potently and safely eliminate CAFs from the TME.

Bacteria‐based cancer therapy offers a unique and powerful solution [12, 13]. Engineered bacteria can act as autonomous therapeutic vehicles, using their natural motility to deeply penetrate tumors and selectively colonize the immunosuppressive TME [14]. Furthermore, they can be programmed to function as localized “bioreactors” to produce and deliver potent payloads directly at the tumor site [15, 16].

Here, we developed CAT‐BLAST (CAF‐Targeting Bacteria for Localized And Suppressive Therapy) by engineering a novel bacterium capable of (1) selectively targeting and binding to FAP‐positive CAFs and (2) inducing localized apoptosis to dismantle the protective CAF barrier and nearby cancer cells, aiming to overcome therapeutic resistance and enhance anti‐tumor efficacy.

## ENGINEERING AN OPTIMAL BACTERIAL CHASSIS FOR ENHANCED SAFETY AND POTENT EXPRESSION

To develop a safe and effective bacterial chassis, we began with two candidate strains: *E. coli* K12 MG1655 and BL21(DE3). Our first priority was to enhance safety. By deleting the entire *fimA*‐*H* operon responsible for type I fimbriae, we created two new strains, EcM1 and EcB1 (Figure S1), which showed a significantly reduced non‐specific binding to both iBCAFs (immortalized breast CAFs) and MCF‐7 breast cancer cells without affecting bacterial growth (Figure 1A,B and Figure S2A).

**Figure 1:**
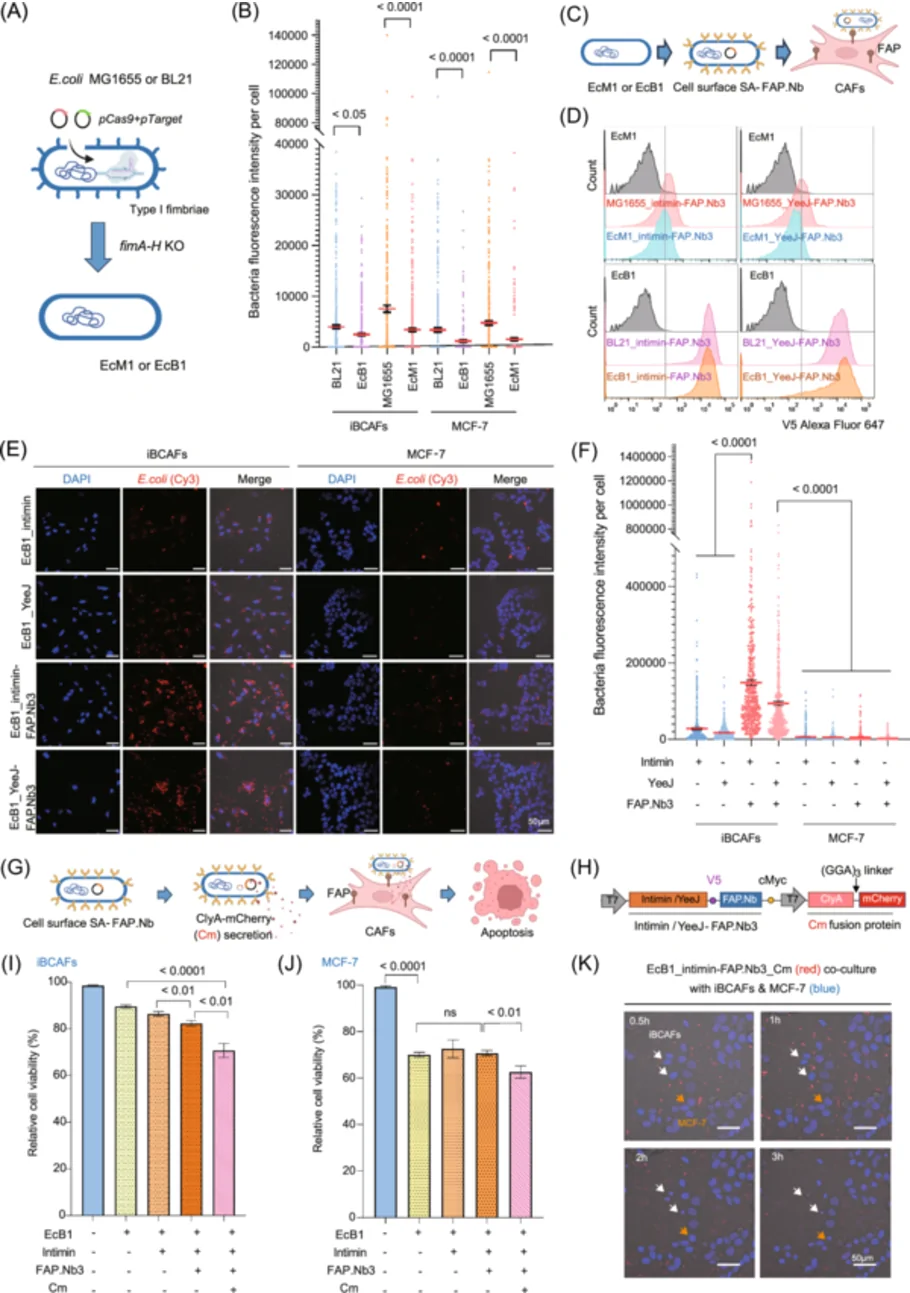
Design and in vitro validation of the CAT‐BLAST platform for targeted cell killing. (A) Schematic of the generation of safer *E. coli* chassis by deleting the *fimA‐H* operon. (B) Quantification of bacterial adhesion from images shown in Figure S2A, demonstrating reduced non‐specific binding of the EcM1 and EcB1 strains. (C) Schematic illustrating the synthetic adhesin (SA) strategy for targeting FAP on the surface of CAFs. (D) Flow cytometry analysis comparing surface expression of the SA‐FAP.Nb3 construct in the indicated bacterial strains. (E) Representative confocal images showing specific enrichment of FAP‐targeting bacteria (red) on FAP‐positive iBCAFs versus FAP‐negative MCF‐7 cells. Nuclei are stained with DAPI (blue). Scale bars, 50 µm. (F) Quantification of bacterial enrichment from (E), measured as fluorescence intensity per cell. (G) Schematic of the complete CAT‐BLAST system, armed with the secretable ClyA‐mCherry (Cm) cytotoxin. (H) Diagram of the pETDuet vector encoding both the SA and the Cm payload. Cell viability quantification from the apoptosis assay for iBCAFs (I) and MCF‐7 cells (J) following co‐culture with the indicated bacteria. (K) Time‐lapse confocal microscopy of a mixed co‐culture treated with CAT‐BLAST. White arrows indicate apoptotic iBCAFs (targeted cells); orange arrows indicate apoptotic MCF‐7 cells (bystander killing). The two cell types were distinguished in post‐acquisition analysis by their distinct morphologies. Scale bars, 50 µm. Data in (B), (F), (I), and (J) are mean ± SEM from *n* = 3 biologically independent experiments. Statistical significance was determined by unpaired, two‐tailed Student's *t*‐test (ranges of *p* values are labeled; ns, not significant).

Next, to engineer CAF‐specific targeting, we designed synthetic adhesins (SAs) by fusing a membrane anchor (either intimin or YeeJ) to a high‐affinity FAP‐targeting nanobody (Figure 1C and Figure S2B). After comparing three candidate FAP nanobodies [17], we identified nanobody‐3 (Nb.3) as the most effective for targeting iBCAFs and selected it for all subsequent constructs (Figure S3A,B). With the optimal SA designed, we compared its expression in both chassis. We observed that EcB1 consistently demonstrated significantly higher and more stable SA expression than EcM1 (Figure 1D and Figure S2C). Based on these findings, we selected EcB1 as the optimal chassis for the subsequent development of the CAT‐BLAST system.

## ENGINEERED BACTERIA ACHIEVE HIGH‐AFFINITY, FAP‐SPECIFIC TARGETING OF CAFs

We next sought to validate FAP‐targeting specificity of our engineered bacteria by co‐culturing them with both FAP‐positive iBCAFs and FAP‐negative MCF‐7 cells (Figure S4). As expected, the bacteria bearing the selected FAP nanobody demonstrated significantly higher binding and enrichment around iBCAFs compared to MCF‐7 cells (Figure 1E,F and Figure S5). In contrast, control bacteria lacking the FAP nanobody showed minimal binding, confirming that targeting was driven by the FAP nanobody. Furthermore, the modularity and specificity of our synthetic adhesin design were confirmed using a control bacterium engineered to target GFP, which bound exclusively to EGFP‐expressing HeLa cells (Figure S6), a result consistent with a previous study using a similar approach [18].

Finally, we performed FAP‐knockout in iBCAFs using CRISPR‐Cas9 (Figure S7A,B), and revealed that the adhesion of our engineered bacteria to these FAP‐depleted cells was dramatically reduced (Figure S7D–F), validating that the targeting of CAT‐BLAST is strictly dependent on the presence of FAP on the CAF surface.

## SECRETION OF ClyA TOXIN ENABLES POTENT KILLINGS OF CAFs AND TUMOR CELLS

To augment the tumor‐killing capacity of our CAF‐targeting bacteria, we further engineered them to express pore‐forming cytotoxic protein ClyA (Figure 1G). ClyA is a well‐characterized α‐pore‐forming toxin that induces apoptosis by directly oligomerizing on the target cell membrane to form a pore complex in a cholesterol‐dependent manner [19, 20]. For secretion and fluorescent tracking, ClyA was fused to a signal peptide and an mCherry tag, creating the “Cm” payload (Figure 1H). We confirmed the payload's potent expression and successful secretion from the EcB1 chassis and verified that the secreted toxin was highly bioactive, inducing significant apoptosis in iBCAFs (Figure S8).

In co‐culture experiments, the complete CAT‐BLAST system (EcB1_intimin‐FAP.Nb3_Cm) significantly enhances apoptosis in both iBCAFs and MCF‐7 compared to bacteria lacking the Cm payload (Figure 1I,J). Crucially, in a mixed co‐culture of both cell types, this cytotoxicity was not restricted to the targeted iBCAFs, as we also observed potent bystander killing of the adjacent, FAP‐negative MCF‐7 cancer cells (Figure 1K). These findings validate our design strategy and provide a strong rationale for the anticipated in vivo therapeutic efficacy of CAT‐BLAST.

## CAT‐BLAST DEMONSTRATES ROBUST TARGETING ACROSS DIVERSE TUMOR MODELS

To assess the CAT‐BLAST platform in vivo, we first confirmed that our selected human FAP‐targeting nanobody (FAP.Nb3) cross‐reacts with mouse FAP, a conclusion supported by their high sequence similarity and by computational modeling (Figures S9 and S10).

We then sought to validate the platform's targeting capabilities across a wide range of tumors, including syngeneic colorectal, melanoma and human xenografts of breast, bladder, lung, prostate, and pancreatic cancer. After confirming all selected tumor models were FAP‐positive via immunohistochemistry (Figure S11), we performed intravenous (IV) injections. In all models tested, the FAP‐targeting bacteria (EcB1_intimin‐FAP.Nb3) successfully co‐localized with FAP‐rich regions, demonstrating robust targeting specificity across diverse TME (Figure S12).

A more detailed analysis was then performed in the MC38 colorectal model. Following IV injection, the FAP‐targeting bacteria (EcB1_intimin‐FAP.Nb3) demonstrated significantly enhanced tumor‐specific colonization compared to all non‐targeting controls (Figure S13A,B). Organ biodistribution analysis showed that while the IV injected bacteria were largely cleared by the liver and spleen, EcB1_intimin‐FAP.Nb3 successfully localized to the tumor periphery, a region rich in mouse CAFs (Figure S13C). Further analysis revealed that EcB1_intimin‐FAP.Nb3 achieved both faster and stronger colonization kinetics than the control strain (Figure S13D,E). Importantly, as a functional consequence of this enhanced targeting, EcB1_intimin‐FAP.Nb3 also exhibited stronger tumor suppression (Figure S13F). Biosafety evaluations confirmed that the treatment was transient and well‐tolerated, with bacteria cleared from feces after 7 days and no significant impact on mice weight (Figure S13G,H).

## CAT‐BLAST POTENTLY SUPPRESSES COLORECTAL TUMOR AND MELANOMA IN VIVO

To further evaluate the therapeutic potential of the CAT‐BLAST system, we next assessed its tumor suppression efficacy following direct intratumoral administration in the MC38 model (Figure 2A). In vivo imaging confirmed that the bacteria remained specifically localized within the tumor 24 h post‐injection, with no detectable spread to healthy tissues (Figure 2B,C). Notably, EcB1_intimin‐FAP.Nb3, even with intratumoral injection, preferentially accumulated at the tumor periphery. Histological analysis of tumor sections corroborated these findings, showing significant morphological alterations in FAP+ cells following treatment with both the targeting (EcB1_intimin‐FAP.Nb3) and the therapeutic (EcB1_intimin‐FAP.Nb3_Cm) bacteria (Figure 2D), resulting in significant tumor destruction (Figure 2E). This potent local therapy effectively suppressed tumor growth and was well‐tolerated, confirming the platform's robust efficacy and favorable safety profile (Figure 2F,G).

**Figure 2:**
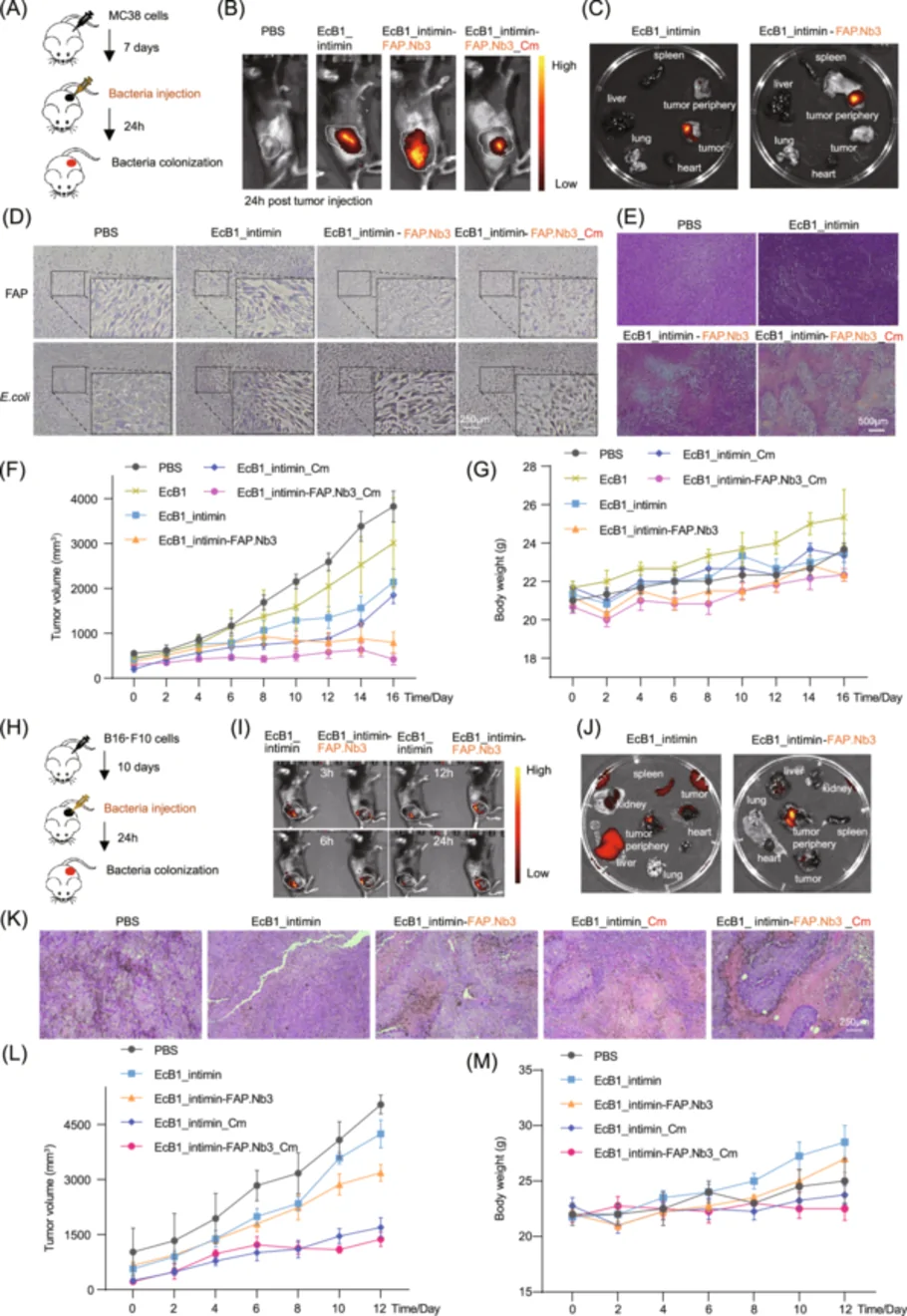
CAT‐BLAST achieves potent tumor suppression in murine colorectal and melanoma models. (A) Schematic of the intratumoral treatment schedule for the MC38 colorectal cancer model. (B) Representative in vivo imaging of tumor‐bearing mice 24 h after injection with DiR‐labeled bacteria, showing specific tumor accumulation. (C) Ex vivo imaging of dissected organs confirms tumor‐specific localization of FAP‐targeting bacteria. (D) IHC staining of MC38 tumor sections confirms co‐localization of *E. coli* with FAP‐expressing regions. Scale bars, 250 µm. (E) H&E staining of MC38 tumor sections reveals significant tumor destruction and necrosis in CAT‐BLAST treated groups. Scale bars, 500 µm. Tumor growth curves (F) and corresponding mouse body weights (G) demonstrate the therapeutic efficacy and tolerability of CAT‐BLAST in the MC38 model (*n* = 3). (H) Schematic of the treatment schedule for the B16‐F10 melanoma model. (I) Time‐course in vivo imaging shows superior retention of FAP‐targeting bacteria within B16‐F10 tumors. (J) Ex vivo imaging of organs from B16‐F10 mice confirms enhanced tumor retention. (K) H&E staining reveals distinct tumor destruction in B16‐F10 tumors treated with CAT‐BLAST. Scale bars, 250 µm. Tumor growth curves (L) and mouse body weights (M) show significant tumor suppression and good tolerability in the B16‐F10 model (*n* = 3).

We then assessed CAT‐BLAST's therapeutic efficacy in a B16‐F10 melanoma model, which is often used to represent hard‐to‐treat “cold” tumor (Figure 2H). Unlike in the MC38 model where non‐targeting bacteria remained tumor‐localized after intratumoral injection, in vivo imaging of B16‐F10 tumors showed a different phenomenon. While FAP‐targeting bacteria were mostly retained within the tumor, the non‐targeting control strain quickly dispersed to distant organs (Figure 2I,J). We hypothesize this difference is due to the less structured, more fluid‐like nature of the B16 melanoma. Consistent with the colorectal model, the complete CAT‐BLAST system (EcB1_intimin‐FAP.Nb3_Cm) caused distinct tumor destruction, significantly suppressed melanoma growth, with only minimal impact on body weight in treated mice (Figure 2K–M).

While our initial biosafety evaluation, including stable body weights (Figure 2G,M and Figure S13H), transient fecal shedding (Figure S13G), and the H&E staining of main organs (Figure S14) indicates that the CAT‐BLAST platform is well‐tolerated, we acknowledge that a comprehensive preclinical safety assessment, including hematology, blood biochemistry, and histopathological analysis of major organs, will be an essential component of future translational studies.

In summary, we have developed CAT‐BLAST, an engineered bacterium designed to remodel the TME by dismantling CAFs. By combining a FAP‐specific adhesin for precision targeting with a secretable ClyA toxin, CAT‐BLAST effectively kills both CAFs and adjacent tumor cells. This dual‐action platform demonstrated robust tumor colonization and potent anti‐tumor efficacy across diverse in vivo models, offering a new strategy for overcoming stromal barriers in cancer therapy.

## AUTHOR CONTRIBUTIONS

**Mengdi Xu**: Methodology; investigation; data curation; formal analysis; visualization; writing—original draft; writing—review and editing. **Ehsan Hashemi**: Methodology; investigation; data curation; formal analysis; writing—original draft; writing—review and editing. **Hui Gao**: Methodology; supervision; writing—original draft. **Qumar Zaman**: Methodology; Writing—original draft. **Yi Ma**: Supervision; resources. **Jufang Wang**: Supervision; resources. **Wenjun Mao**: Resources. **Zhuobin Liang**: Conceptualization; investigation; methodology; supervision; funding acquisition; project administration; writing—original draft; writing—review and editing. All authors have read the final manuscript and approved it for publication.

## CONFLICT OF INTEREST STATEMENT

Z.L. and M.X. are inventors on a granted patent filed by Shenzhen Bay Laboratory (CN118995547B) that covers engineered bacteria and their application in tumor therapy. Other authors declare no conflicts of interest.

## ETHICS STATEMENT

The ethics application (No. AELZB202301) was approved by the Institutional Animal Care and Use Committee (IACUC) of the Animal Experiment Center of Shenzhen Bay Laboratory.

## Supporting information


**Figure S1.** Generation of *fimA‐H* operon knockout strains.
**Figure S2.** Reduced non‐specific binding and synthetic adhesin (SA) expression.
**Figure S3.** Comparison of FAP nanobody targeting affinity.
**Figure S4.** FAP expression in iBCAFs but not MCF‐7 cells.
**Figure S5.** High‐content imaging quantification of bacterial enrichment.
**Figure S6.** Validation of synthetic adhesin modularity and specificity.
**Figure S7.** FAP‐dependent targeting validated by CRISPR‐Cas9 knockout.
**Figure S8.** Validation of ClyA‐mCherry (Cm) secretion and bioactivity.
**Figure S9.** Protein sequence alignment of human and mouse FAP.
**Figure S10.** Predicted structural interaction between FAP.Nb3 and FAP.
**Figure S11.** FAP expression across diverse tumor models.
**Figure S12.** Broad *in vivo* targeting efficacy of CAT‐BLAST.
**Figure S13.** *In vivo* targeting kinetics and biosafety evaluation in the MC38 model.
**Figure S14.** Histological analysis of major organs after systemic bacterial administration.
**Supplementary Table 1.** Sequences of key genes and primers used in this study.

**Supplementary File 1.** Source Data.

## Data Availability

The data that support the findings of this study are available in the supplementary material of this article. All data are available in the main text, supplementary figures, supplementary table and source data. Supplementary materials (figures, tables, graphical abstract, slides, videos, Chinese translated version, and update materials) may be found in the online DOI or iMeta Science http://www.imeta.science/.
