# Supplementary material for: CAT‐BLAST: Engineered bacteria for robust targeting and elimination of cancer‐associated fibroblasts
Source: Imeta. 2026 Jan 5;5(1):e70102. doi: 10.1002/imt2.70102 (PMC13726584; doi:10.1002/imt2.70102)
Supplement: Supplementary file 1 [file IMT2-5-e70102-s001.docx]

**Supporting information to CAT-BLAST: Engineered Bacteria for Robust Targeting and Elimination of Cancer-Associated Fibroblasts**

Mengdi Xu ^1, 2^, Ehsan Hashemi ^2^, Hui Gao ^2^, Qumar Zaman ^2^, Yi Ma ^1^, Jufang Wang ^1^, Wenjun Mao ^3^, Zhuobin Liang ^2^*

^1^ School of Biology and Biological Engineering, South China University of Technology, Guangzhou, 510006, China.

^2^ Institute of Molecular Physiology, Shenzhen Bay Laboratory, Shenzhen, 518132, China.

^3^ Department of Cardiothoracic Surgery, The Affiliated Wuxi People's Hospital of Nanjing Medical University, Wuxi, Jiangsu, 214023 China.

* Correspondence: [zhuobin.liang@outlook.com](mailto:zhuobin.liang@outlook.com) (Zhuobin Liang)

**METHODS**

**Bacterial Strains and Cell Culture**

*Escherichia coli* K12 MG1655 was from our laboratory collection. *E. coli* BL21(DE3) and DH5α were purchased from AlphalifeBio (Shenzhen, China). All bacterial strains were cultured in Luria-Bertani (LB) broth or on LB agar (Sangon Biotech, Shanghai, China) at 37°C with shaking, supplemented with kanamycin (50 µg/mL), ampicillin (100 µg/mL), or spectinomycin (50 µg/mL) as required.

Immortalized human breast cancer-associated fibroblasts (iBCAFs) were purchased from iCellbioscience (Shanghai, China) and cultured in RPMI 1640 medium (Gibco). Human breast cancer cells (MCF-7), provided by Bayray Innovation Center (Shenzhen, China), were cultured in EMEM (NEXELL) supplemented with 0.01 mg/mL recombinant human insulin (YEASEN). Human cervical cancer cells (HeLa) were cultured in DMEM (Gibco). All mammalian cell culture media were supplemented with 10% fetal bovine serum (FBS, TransGen) and 1% penicillin-streptomycin solution (Sangon). Cells were maintained at 37°C in a humidified 5% CO₂ incubator.

**Plasmid and Strain Construction**

***fimA-H* operon deletion.** To generate safer bacterial chassis, the entire *fimA-H* operon was deleted from *E. coli* K12 MG1655 and BL21(DE3) using a pCas9/pTarget2N20 CRISPR-Cas9 system. Two sgRNAs were designed to target the *fimB* and *fimH* genes at the start and end of the operon, respectively (Supplementary Table 1). A 1000 bp donor DNA fragment consisting of 500 bp homology arms flanking the operon was synthesized. The pCas9 plasmid was first transformed into the parent strains. Cas9 expression was induced with 10 mM arabinose for 1.5 h before the cells were made electrocompetent. The pTarget2N20 plasmid (containing the sgRNAs) and the donor fragment were co-electroporated (2500 V, 0.5 ms; Bio-Rad Gene Pulser Xcell™). Clones were selected on LB agar containing kanamycin and spectinomycin. Successful knockout was confirmed by PCR verification, Sanger sequencing of the genomic locus, and antibiotic sensitivity. The pCas9 and pTarget2N20 plasmids were subsequently cured using their encoded counter-selection mechanisms (*sacB*-mediated sucrose sensitivity and rhamnose-induced pMB1 sgRNA expression, respectively).

**Synthetic adhesin (SA) construction.** Plasmids for expressing SAs were constructed in a pET28a(+) vector backbone. Two membrane anchors were used: intimin, derived from enteropathogenic *E. coli* and codon-optimized for expression in BL21(DE3), and YeeJ, an inverse autotransporter native to *E. coli* K12 MG1655. The coding sequences for three high-affinity FAP-targeting nanobodies (FAP.Nb) were codon-optimized and inserted into the NheI and BamHI sites of the pET vector, replacing a 6×His tag. The final SA constructs included an N-terminal signal peptide, a peptidoglycan-binding LysM domain, the β-barrel membrane anchor, and the surface-exposed FAP.Nb flanked by V5 and c-Myc epitope tags to facilitate detection. All gene sequences are provided in Supplementary Table 1.

**Cytotoxin payload construction.** To create the therapeutic and imaging strains, a pETDuet vector was used. The SA expression cassette (pET_Intimin-FAP.Nb3 or pET_YeeJ-FAP.Nb3) was maintained under the first T7 promoter. The coding sequence for cytolysin A (*ClyA*) from *Salmonella typhimurium* was fused to mCherry via a (GGA)₃ linker to create the Cm payload. This *Cm* fusion gene was placed under the control of a second T7 promoter. A control plasmid expressing mCherry without *ClyA* was also constructed for *in vivo* bacterial detection.

***In Vitro* FAP Depletion in iBCAFs.** FAP-depleted iBCAFs were generated using a lentivirus-based CRISPR-Cas9 approach. Two LentiCRISPRv2 vectors were constructed, each expressing a unique FAP-targeting sgRNA and either a blasticidin or puromycin resistance gene (Supplementary Table 1). Lentiviral particles were produced by co-transfecting HEK293T cells with the two sgRNA vectors, psPAX2, and pMD2.G. At 48 h post-transfection, the virus-containing supernatant was collected, filtered (0.45 µm), and supplemented with 8 µg/mL polybrene. iBCAFs were then transduced with the mixed lentiviral suspension by centrifugation at 1,500 rpm for 15 min. Starting at 72 h post-transduction, the cells were cultured under dual selection with blasticidin (10 µg/mL) and puromycin (4 µg/mL) until all non-transduced cells were eliminated. Successful FAP depletion was confirmed by RT-qPCR and Western blotting. As single-cell cloning of FAP-depleted iBCAFs proved difficult due to poor growth, all subsequent experiments were performed using the stable FAP-depleted cell population.

**Gene and Protein Expression Analysis**

**RT-qPCR.** Total RNA was extracted from wild-type and FAP-depleted iBCAFs using the RNAeasy™ Animal RNA Isolation Kit (Beyotime). 500 ng of RNA was reverse transcribed into cDNA using the Hifair® III 1st Strand cDNA Synthesis SuperMix (YEASEN). qPCR was performed in triplicate using SYBR Green master mix (YEASEN) in a 20 µL reaction volume containing 800 ng of cDNA. Thermal cycling conditions were: 95°C for 10 min, followed by 40 cycles of 95°C for 15 s and 60°C for 1 min. FAP mRNA levels were normalized to *GAPDH*, and relative expression was calculated using the 2^-ΔΔCT^ method.

**Western Blotting.** Cells were lysed in ice-cold RIPA buffer (Beyotime), and protein concentration was determined using a BCA Protein Assay Kit (Beyotime). 2 µg of protein per sample was denatured at 95°C for 10 min in 5× SDS-PAGE loading buffer (Genstar), resolved by SDS-PAGE, and transferred to a nitrocellulose membrane (Bio-Rad). Membranes were blocked for 1 h in 5% (w/v) non-fat milk in TBST and incubated overnight at 4°C with the following primary antibodies: anti-FAP (1:1000, Cell Signaling Technology), anti-β-actin (1:10000, Abclonal), or anti-Na+/K+ ATPase (1:5000, Proteintech). After washing, membranes were incubated for 1 h with HRP-conjugated Goat Anti-Rabbit IgG secondary antibody (1:2000, Abcam). Blots were visualized using ECL reagent (Yamei) and imaged on a Bio-Rad ChemiDoc MP Imaging System.

**Cellular Assays**

**Immunofluorescence.** iBCAFs (2×10⁵) or MCF-7 cells (3×10⁵) were seeded on 8 mm glass slides in 24-well plates and cultured overnight. Cells were fixed with 4% paraformaldehyde (PFA) for 15 min, permeabilized with 0.3% Triton X-100 for 10 min, and blocked with 10% normal goat serum for 30 min. Slides were incubated overnight at 4°C with anti-FAP antibody (1:500, Cell Signaling Technology), followed by incubation with FITC-conjugated Goat Anti-Rabbit IgG secondary antibody (1:100, Abclonal) for 1 h at 37°C. Nuclei were counterstained with 1 µg/mL DAPI. Slides were mounted with antifade mounting medium (Beyotime) and imaged on a Nikon Confocal Laser Scanning Microscope.

***In vitro* adhesion and enrichment quantification.** Bacterial strains were grown to an OD600 of ≈0.7, and protein expression was induced with 0.05 mM IPTG for 8-12 h. Bacteria were washed and labeled first with a polyclonal anti-*E. coli* antibody (1:1000, Invitrogen) for 45 min, followed by a Cy3-conjugated secondary antibody (1:500, Abclonal) for 45 min. Labeled bacteria were resuspended in cell culture medium and added to cells seeded on glass slides at a multiplicity of infection (MOI) of 300:1. After 1 h of co-incubation, cells were washed five times with PBS, fixed with 4% PFA, and stained with DAPI. For quantification, over 5,000 cells per sample were imaged using an Opera Phenix Plus high-content screening system. The system identified individual cells (DAPI channel) and quantified the associated bacterial fluorescence (Cy3 channel) within a defined perimeter. The mean fluorescence intensity per cell was calculated from a random selection of 500 cells per group.

**Cell apoptosis assays.** For the bacteria-free medium assay, bacteria were cultured with IPTG, and the culture medium was collected and filter-sterilized (0.45 µm). iBCAFs were treated for 16 h with a 1:1 mixture of the filtered bacterial medium and fresh RPMI 1640, with 1M HEPES buffer added to maintain pH. For the bacterial co-culture assay, IPTG-induced bacteria were added to iBCAFs or MCF-7 cells at an MOI of 300:1 in medium containing HEPES, IPTG, and 15 µg/mL chloramphenicol to prevent bacterial overgrowth. After 4 h of co-culture, cells from both assays were harvested, stained with an Annexin V-APC/PI Apoptosis Detection Kit (Vazyme), and analyzed by flow cytometry.

**Animal Studies**

**Ethics statement.** All animal experiments were approved by the Institutional Animal Care and Use Committee (IACUC) of the Animal Experiment Center of Shenzhen Bay Laboratory (No. AELZB202301).

**Tumor models.** 6-8 week old C57BL/6N and BALB/c nude mice were obtained from Gempharmatech Co., Ltd. and acclimated for 2 weeks. Syngeneic MC38 and B16-F10 tumors were established by subcutaneously inoculating 1×10⁶ cells mixed 1:1 with Matrigel into the right flank of C57BL/6N mice. A co-injection xenograft model was established by subcutaneously injecting a mixture of 1×10⁷ iBCAFs and 1×10⁷ MCF-7 cells with Matrigel (1:1) into BALB/c nude mice. Additional human xenograft models (MCF-7, 5637, A549, LNCaP, PANC-1) were purchased from Royo Biotech (Nanchang, China). Experiments commenced when tumors reached a volume of 200-600 mm³. Tumor volume was calculated using the formula: Volume = 0.5 × Length × Width².

***In vivo* biodistribution and therapeutic efficacy.** Bacteria were cultured and induced with IPTG as described above. For imaging, 1×10⁹ CFU of bacteria were labeled with 2 µM DiR dye for 30 min at room temperature. Mice were administered 100 µL of PBS containing 1×10⁸ CFU of bacteria via tail vein (IV) or direct intratumoral (IT) injection. Bacterial distribution was monitored using an IVIS Spectrum 3D *in vivo* imaging system (Perkin Elmer). For efficacy studies, tumor volume and body weight were measured daily.

**Biosafety evaluation.** To monitor bacterial shedding, fresh feces (5 mg) were collected daily from each mouse for one week, resuspended in 200 µL of water, and plated on kanamycin LB agar. Bacterial load was calculated as CFU per gram of feces. To evaluate the potential influence of the administered bacteria on major organs, healthy mice were IV injected with either 100 µL PBS or 1×10⁸ CFU of bacteria. 14 days post-injection, the heart, liver, spleen, lung, and kidney were collected from each group for H&E staining.

**Computational Modeling**

The amino acid sequences of FAP.Nb3 and either human FAP (hFAP) or mouse FAP (mFAP) were submitted to the AlphaFold3 server to predict the structure of their interaction complex. The resulting models were visualized using ChimeraX software (v1.8), and potential hydrogen bonds at the binding interface were analyzed.

**Statistical Analysis**

Data are presented as mean ± SEM. Statistical significance between two groups was determined using an unpaired, two-tailed Student’s t-test. Significance thresholds were set at *p* < 0.05, *p* < 0.01, *p* < 0.001, and *p* < 0.0001.

**SUPPLEMENTARY MATERIALS**

**Figure S1.** Generation of *fimA-H* operon knockout strains.

**Figure S2.** Reduced non-specific binding and synthetic adhesin (SA) expression.

**Figure S3.** Comparison of FAP nanobody targeting affinity.

**Figure S4.** FAP expression in iBCAFs but not MCF-7 cells.

**Figure S5.** High-content imaging quantification of bacterial enrichment.

**Figure S6.** Validation of synthetic adhesin modularity and specificity.

**Figure S7.** FAP-dependent targeting validated by CRISPR-Cas9 knockout.

**Figure S8.** Validation of ClyA-mCherry (Cm) secretion and bioactivity.

**Figure S9.** Protein sequence alignment of human and mouse FAP.

**Figure S10.** Predicted structural interaction between FAP.Nb3 and FAP.

**Figure S11.** FAP expression across diverse tumor models.

**Figure S12.** Broad *in vivo* targeting efficacy of CAT-BLAST.

**Figure S13.** *In vivo* targeting kinetics and biosafety evaluation in the MC38 model.

**Figure S14.** Histological analysis of major organs after systemic bacterial administration.

**Supplementary Table 1.** Sequences of key genes and primers used in this study.

**Supplementary File 1.** Source Data.

**Figure S1. Generation of *fimA-H* operon knockout strains.** (A) Schematic of the CRISPR-Cas9 system, including pCas9 and pTarget2N20 plasmids, used for deleting the *fimA-H* operon in *E. coli*. (B) PCR verification of the successful gene knockout in selected clones of EcB1 and EcM1 using genomic DNA as a template. (C) 24-hour bacterial growth curves (OD600) showing that *fimA-H* deletion does not impair bacterial growth rates compared to wild-type strains. (D) Representative brightfield microscopy images showing the morphology of the indicated bacterial strains.

**Figure S2. Reduced non-specific binding and synthetic adhesin (SA) expression.** (A) Representative confocal microscopy images of bacterial adhesion to iBCAFs (FAP-positive) and MCF-7 cells (FAP-negative), illustrating the reduction in non-specific binding of EcM1 and EcB1 strains. (B) Diagram of the intimin- and YeeJ-based SA constructs, showing the key domains including the surface-displayed FAP nanobody flanked by V5 and cMyc epitope tags. (C) Flow cytometry quantification of the percentage of bacteria expressing surface SAs, confirming high expression levels in the EcB1 chassis.

**Figure S3. Comparison of FAP nanobody targeting affinity.** (A) Representative confocal images showing the enrichment of bacteria expressing one of three different FAP nanobody candidates around iBCAFs. (B) Quantification of bacterial enrichment for each nanobody construct, measured as fluorescence intensity per cell, identifying FAP.Nb3 as the top-performing candidate for subsequent experiments.

**Figure S4. FAP expression in iBCAFs but not MCF-7 cells.** (A) Immunofluorescence staining confirms FAP expression (green) on the surface of iBCAFs, which is absent in MCF-7 cells. (B) Western blot analysis of whole protein (WP), intracellular protein (IP), and membrane protein (MP) fractions confirming that FAP is primarily a membrane-bound protein in iBCAFs.

**Figure S5. High-content imaging quantification of bacterial enrichment.** (A) Representative images captured by the Opera Phenix Plus high-content screening system, showing bacteria (red) enriched on iBCAFs (blue). (B) Quantification of bacterial fluorescence intensity per cell from high-content screening, confirming the specific and significant binding of FAP-targeting bacteria.

**Figure S6. Validation of synthetic adhesin modularity and specificity.** (A) Schematic of the GFP-targeting SA construct used as a specificity control. (B) Flow cytometry analysis confirming the surface expression of the GFP-targeting SA in engineered bacteria. (C) Confocal images showing that GFP-targeting bacteria (red) bind exclusively to HeLa cells expressing surface EGFP (green) and not to wild-type HeLa cells, demonstrating the platform's modularity.

**Figure S7. FAP-dependent targeting validated by CRISPR-Cas9 knockout.** (A, B) RT-qPCR (A) and Western blot (B) analysis confirming significant depletion of FAP expression in the iBCAF cell population following CRISPR-Cas9 knockout. (C) Brightfield images showing the morphology of wild-type and FAP-depleted iBCAF cells. (D, E) Confocal (D) and high-content screening (E) images showing dramatically reduced adhesion of FAP-targeting bacteria to the FAP-depleted iBCAFs. (F) Quantification of bacterial enrichment, validating that targeting is strictly dependent on FAP expression.

**Figure S8. Validation of ClyA-mCherry (Cm) secretion and bioactivity.** (A) Cm expression kinetics over 16 hours, measured by mCherry fluorescence, showing induction by IPTG. (B, C) Flow cytometry analysis (B) and corresponding quantification (C) demonstrating high-level co-expression of the SA (V5-Alexa Fluor 647 signal) and the Cm payload (mCherry signal). The intimin anchor yields a significantly higher percentage of double-positive bacteria than the YeeJ anchor and was therefore selected for all subsequent constructs. (D) Measurement of mCherry fluorescence in the culture supernatant, confirming secretion of the Cm payload from the bacteria. (E) Workflow for the bioactivity assay using 0.45µm-filtered, bacteria-free medium. (F, G) Flow cytometry apoptosis assay (F) and corresponding cell viability quantification (G) showing that the secreted Cm payload is bioactive and induces potent apoptosis in iBCAFs.

**Figure S9. Protein sequence alignment of human and mouse FAP.** Sequence alignment generated by Clustal-Omega showing high amino acid identity (89.4%) and similarity (94.2%) between human (hFAP) and mouse (mFAP) FAP, supporting the cross-reactivity of the FAP.Nb3 nanobody.

**Figure S10. Predicted structural interaction between FAP.Nb3 and FAP.** Structural models generated by AlphaFold3 showing the predicted hydrogen bond interactions at the binding interface between the FAP.Nb3 nanobody and (A) human FAP (hFAP) and (B) mouse FAP (mFAP).

**Figure S11. FAP expression across diverse tumor models.** IHC staining confirms FAP protein expression in a panel of murine syngeneic (MC38, B16-F10) and human xenograft (iBCAFs&MCF-7, MCF-7, 5637, LNCaP, PANC-1, A549) tumor models.

**Figure S12. Broad *in vivo* targeting efficacy of CAT-BLAST.** (A) Schematic of the intravenous (IV) injection protocol used for assessing targeting. (B-I) Representative immunofluorescence images of tumor sections from eight different tumor models, 48 hours after IV injection. Images show robust co-localization of FAP-targeting bacteria (red) with FAP-positive tumor regions (green).

**Figure S13. *In vivo* targeting kinetics and biosafety evaluation in the MC38 model.** (A) Schematic of the IV injection protocol. (B, C) *In vivo* (B) and *ex vivo* (C) imaging 24 hours post-injection, showing enhanced tumor colonization by FAP-targeting bacteria compared to controls. (D) Time-course *in vivo* imaging demonstrating the colonization kinetics over 24 hours. (E) *Ex vivo* image of a dissected tumor showing bacterial localization concentrated at the FAP-rich tumor periphery. While the bacterial signal in the tumor is masked by the much brighter signal from clearance organs in the composite image (C), its specific localization is clearly visible when the tumor is imaged separately with optimized exposure settings (E). (F, H) Tumor growth curves (F) and mouse body weights (H) following IV injection of targeting bacteria without payload. (G) Bacterial clearance kinetics measured from feces, confirming transient bacterial presence.

**Figure S14. Histological analysis of major organs after systemic bacterial administration.** Healthy mice were intravenously injected with PBS or 1×10⁸ CFU of the indicated bacterial strains. The heart, liver, spleen, lung, and kidney were collected 14 days post-injection for H&E staining.

**Supplementary Table 1. Sequences of key genes and primers used in this study**

| Type | Name | Sequence (5’-3’) |
| --- | --- | --- |
| gene | intimin | atgattacccatggttgttatacccgtacgcgtcataaacacaaactgaaaaagaccctgattatgctgagcgcaggtctgggtctgtttttctatgttaatcagaatagctttgccaacggcgagaactatttcaaactgggtagcgatagcaaactgctgacccatgatagttatcagaatcgcctgttttataccctgaaaaccggtgaaaccgttgcagatctgagcaaaagccaggatattaatctgagcaccatttggagcctgaacaaacatctgtatagcagcgaaagcgaaatgatgaaagcagcacctggtcagcagattattctgccgctgaaaaagctgccgtttgaatatagcgcactgccgctgctgggtagtgcaccgctggttgcagccggtggtgttgcaggtcataccaataaactgaccaaaatgagtccggatgtgaccaaaagcaatatgaccgatgataaagcactgaattatgcagcacagcaggcagcaagcctgggtagccagctgcagagccgtagcctgaatggtgattatgcaaaagataccgcactgggtattgcaggtaatcaggcaagcagtcagctgcaggcctggctgcagcattatggcaccgcagaagttaatctgcagagcggtaataactttgatggtagcagcctggattttctgctgcctttttatgatagcgagaaaatgctggcatttggtcaggttggtgcacgttatattgatagccgttttaccgcaaatttaggtgcaggtcagcgtttctttctgcctgcaaatatgctgggttataacgtgtttatcgatcaggattttagcggtgataacacccgtttaggtattggtggtgaatattggcgtgattactttaaaagcagcgtgaatggttattttcgtatgagcggttggcatgagagctataacaaaaaggattatgatgaacgtccggcaaacggttttgatattcgctttaatggttactgccgagctatccggcactgggtgcaaaactgatttatgaacagtattatggcgataacgtggccctgtttaatagcgataaactgcagagcaatccgggtgcagcaaccgttggtgtgaactataccccgattccgctggttaccatgggcattgattatcgtcatggcaccggtaatgaaaacgatctgctgtatagtatgcagttccgctatcagtttgataaaagctggtcccagcagattgaaccgcagtatgttaatgaactgcgtaccctgagcggtagccgttatgatctggttcagcgcaataataacatcatcctggaatacaaaaagcaggacattctgagcctgaatattccgcatgatattaatggcaccgaacatagcacccagaaaattcagctgattgtgaaaagcaaatatggcctggatcgtattgtttgggatgatagtgcactgcgtagccaaggtggtcagattcagcatagcggtagtcagagcgcacaggattatcaggcaattctgccagcatatgttcaaggtggtagcaacatctataaagttaccgcacgtgcatatgatcgcaatggtaatagcagcaataatgttcagctgacaattaccgttctgagcaatggtcaggtggttgatcaggtgggtgttaccgattttacagccgataaaaccagcgcaaaagcagataatgccgataccattacctataccgcaaccgttaaaaagaatggtgttgcccaggcaaatgttccggttagctttaacattgttagcggtacagcgaccctgggtgccaatagcgcaaaaaccgatgcaaatggtaaagcaaccgtgacactgaaaagcagtaccccaggccaggttgttgttagtgccaaaaccgcagaaatgaccagcgcactgaatgcaagcgcggttatattttttgatatcccaagcttgggtaatggtaagcctatccctaaccctctcctcggtctcgattctacgctgccggctagccggcatcatcaccatcaccattcgcggatccgcgtcgagcagaaactcatctctgaagaggatctgtag |
| gene | YeeJ | atgggaattaaactacgccgcttaactgcgggtatctgtctgataactcaacttgcgttccctatggctgcggcagcacaaggtgtggtaaacgccgcaacccaacaaccagttcctgcacaaattgccattgcaaatgccaatacggtgccctacacccttggagcgttggaatcggcccaaagcgttgccgaacgtttcggtatttcggtggctgagttacgcaaactcaaccagtttcgtacgtttgctcgaggttttgataatgtccgccagggtgatgaactggatgtcccggcacaagttagtgaaaaaaaattaaccccgccgccgggtaatagcagtgacaacctcgagcaacagatagccagtacttcacagcaaatcgggtctctgctcgccgaagatatgaacagcgagcaagcggcaaatatggcgcgtggatgggcctcttctcaggcttcaggcgcaatgacagactggttaagccgcttcggtaccgcaagaatcacgctgggcgtggatgaagattttagcctgaagaactcccagttcgattttctccatccgtggtatgaaacgcctgataatctctttttcagtcagcatactctccatcgtactgacgagcgtacgcagattaacaacggcttaggttggcgtcatttcactcccacatggatgtcgggcatcaacttctttttcgaccacgatcttagccgttaccactcccgcgccggcattggcgcggagtactggcgcgactatctaaaattaagcagtaacggctatttgcgactgaccaactggcgcagcgcacctgaactggacaacgattatgaagcacgcccggccaatggctgggatgtacgcgcagaaagctggctacccgcctggccgcaccttggcggtaaactggtctatgaacagtattatggcgatgaagtggccctgttcgataaagacgatcggcaaagtaatcctcatgccataaccgctggacttaactatacccccttcccgctgatgaccttcagcgcggagcaacgccagggtaaacagggcgaaaatgacacccgttttgccgtcgattttacctggcaacctggcagcgcaatgcagaaacagcttgacccgaatgaagtcgctgcacggcgtagccttgcaggcagccgttatgatctggtggatcgcaacaacaatatcgttctggaatatcgcaaaaaagaactggttcgcctgaccctgacagaccccgtgacagggaagtcaggagaagtgaaatcactggtttcgtcgctacaaaccaaatatgccctgaaaggctataacgtcgaagccaccgcactggaagctgccggtggcaaagtggtcacaacgggtaaagatattctggttaccctgccggcttaccggttcaccagtacgccagaaaccgataacacctggccgattgaagtcaccgccgaagatgtcaaaggcaatttgtcgaatcgtgaacagagcatggttgtcgttcaggcacctacgctaagccagaaagatatcccaagcttgggtaatggtaagcctatccctaaccctctcctcggtctcgattctacgctgccggctagccggcatcatcaccatcaccattcgcggatccgcgtcgagcagaaactcatctctgaagaggatctgtag |
| gene | FAP.Nb1  (2HFA20） | gctagccggcaggttcagctgcaagaaagcggtggtggtctggttcaggcaggcggtagcctgcgtctgagctgtgcagttagcggtcgtctgtttagcgcaaataccatgggttggtatcgtcaggcaccgggtaaacgtcgtgaactggttgccaccattctgagcagcggtagcaccaattatgcagatagcgttaaaggtcgttttaccattagccgtgatgatgccaaaaataccgtttacctgcagatgaatagcctgaaaccggaagataccgcagtgtattattgtaatctggcaccgcctccggaaggttattggggtcagggcacccaggttaccgttagcagccggatcc |
| gene | FAP.Nb2  (2HFA43) | gctagccggcaggttcagctgcaagaaagcggtggtggtctggttcaggcaggcggtagcctgcgtctgagctgtgttgcaagcggcaccatttttagcagcggtgcaatggcaatgggttggtatcgtcaggcaccgggtaaacagcgtgaatgggttgcaggtattaccggtagccgtaccaccacctatgcagatagcgttaaaggtcgttttaccattagccgtgataatgccgaaaataccgtttttctgcagatgaacaatctgaaaagcgaagataccgcagtgtattattgtaatctgtggcctccgagccgtccggatcattggggtcagggcacccaggttaccgttagcagccggatcc |
| gene | FAP.Nb3  (2HFA67) | gctagccggcaggttcagctgcaagaaagcggtggtggtctggttcaggcaggcggtagcctgcgtctgagctgtgttgttagcggtagctttgatagccgtaatgcaatggcatggtatcgtcaggcactgggtaaagaacgtgtttgggttgcaggtattattagtgatggtagcaccaattatgccgatgcagttaaaggtcgttttaccattagccgtgataacgataaaaacaccgtttacctgcagatgaatagcctgaaaccggaagataccgcagtgtattattgtaatgcatggcctccgcgtattggtttaggtagctggggtcagggcacccaggttaccgttagcagccggatcc |
| gene | ClyA | atgattatgaccggaatatttgcagaacaaactgtagaggtagttaaaagcgcgatcgaaaccgcagatggggcattagatctttataacaaatacctcgaccaggtcatcccctggaagacctttgatgaaaccataaaagagttaagccgttttaaacaggagtactcgcaggaagcttctgttttagttggtgatattaaagttttgcttatggacagccaggacaagtattttgaagcgacacaaactgtttatgaatggtgtggtgtcgtgacgcaattactctcagcgtatattttactatttgatgaatataatgagaaaaaagcatcagcccagaaagacattctcattaggatattagatgatggtgtcaagaaactgaatgaagcgcaaaaatctctcctgacaagttcacaaagtttcaacaacgcttccggaaaactgctggcattagatagccagttaactaatgatttttcggaaaaaagtagttatttccagtcacaggtggatagaattcgtaaggaagcttatgccggtgctgcagccggcatagtcgccggtccgtttggattaattatttcctattctattgctgcgggcgtgattgaagggaaattgattccagaattgaataacaggctaaaaacagtgcaaaatttctttactagcttatcagctacagtgaaacaagcgaataaagatatcgatgcggcaaaattgaaattagccactgaaatagcagcaattggggagataaaaacggaaaccgaaacaaccagattctacgttgattatgatgatttaatgctttctttattaaaaggagctgcaaagaaaatgattaacacctgtaatgaataccaacaaagacacggtaagaagacgcttttcgaggttcctgacgtc |
| gene | mCherry | atggtgagcaagggcgaggaggataacatggccatcatcaaggagttcatgcgcttcaaggtgcacatggagggctccgtgaacggccacgagttcgagatcgagggcgagggcgagggccgcccctacgagggcacccagaccgccaagctgaaggtgaccaagggtggccccctgcccttcgcctgggacatcctgtcccctcagttcatgtacggctccaaggcctacgtgaagcaccccgccgacatccccgactacttgaagctgtccttccccgagggcttcaagtgggagcgcgtgatgaacttcgaggacggcggcgtggtgaccgtgacccaggactcctccctgcaggacggcgagttcatctacaaggtgaagctgcgcggcaccaacttcccctccgacggccccgtaatgcagaagaagaccatgggctgggaggcctcctccgagcggatgtaccccgaggacggcgccctgaagggcgagatcaagcagaggctgaagctgaaggacggcggccactacgacgctgaggtcaagaccacctacaaggccaagaagcccgtgcagctgcccggcgcctacaacgtcaacatcaagttggacatcacctcccacaacgaggactacaccatcgtggaacagtacgaacgcgccgagggccgccactccaccggcggcatggacgagctgtacaag |
| Primer | *fimB* sgRNA-F | caagcatattatctcgacttccgg |
| Primer | *fimB* sgRNA-R | aaacccggaagtcgagataatatg |
| Primer | *fimH* sgRNA-F | caaggactatgtcacactgcaacg |
| Primer | *fimH* sgRNA-R | aaaccgttgcagtgtgacatagtc |
| Primer | Left 0.5kb donor-F | caatctcatggcgtaagctg |
| Primer | Left 0.5kb donor-R | agcaatgtcctgtgatttctcgtttttcccttataattac |
| Primer | Right 0.5kb donor-F | gtaattataagggaaaaacgagaaatcacaggacattgct |
| Primer | Right 0.5kb donor-R | cgggattatcagtgctgcaa |
| Primer | Primer A | atcagtgtttacccgccatc |
| Primer | Primer B | cgatttcactatgggtcagga |
| Primer | Primer C | ctggttcagtgccaattcct |
| Primer | Primer D | tgctgaagcagaccatcatc |
| Primer | FAP sgRNA1 F | cacccaataaggcaagcacagcag |
| Primer | FAP sgRNA1 R | aaacctgctgtgcttgccttattg |
| Primer | FAP sgRNA2 F | cacccttgaagggcgtaagacaat |
| Primer | FAP sgRNA2 R | aaacattgtcttacgcccttcaag |
| Primer | ClyA-F | tataagaaggagatatacatatgattatgaccggaatatt |
| Primer | ClyA-R | ttgctcaccattcctcctccgacgtcaggaacctcgaaaa |
| Primer | mcherry-F | ggttcctgacgtcggaggaggaatggtgagcaagggcgag |
| Primer | mcherry-R | gctttgttagcagccggatctcacttgtacagctcgtcca |

“XXX” indicates V5 tag，“ XXX ” indicates 6×His tag，“ XXX ” indicates cMyc tag。
